# Impact of *Rhg1* copy number variation on a soybean cyst nematode resistance transcriptional network

**DOI:** 10.1093/g3journal/jkae226

**Published:** 2024-09-19

**Authors:** Usawadee Chaiprom, Esmaeil Miraeiz, Tong Geon Lee, Jenny Drnevich, Matthew Hudson

**Affiliations:** Department of Crop Sciences, University of Illinois at Urbana-Champaign, Urbana, IL 61801, USA; Illinois Informatics Institute, University of Illinois at Urbana-Champaign, Urbana, IL 61801, USA; Department of Crop Sciences, University of Illinois at Urbana-Champaign, Urbana, IL 61801, USA; Horticultural Sciences Department, University of Florida, Gainesville, FL 32611, USA; High-Performance Biological Computing, Roy J. Carver Biotechnology Center, University of Illinois at Urbana-Champaign, Urbana, IL 61801, USA; Department of Crop Sciences, University of Illinois at Urbana-Champaign, Urbana, IL 61801, USA; Illinois Informatics Institute, University of Illinois at Urbana-Champaign, Urbana, IL 61801, USA; High-Performance Biological Computing, Roy J. Carver Biotechnology Center, University of Illinois at Urbana-Champaign, Urbana, IL 61801, USA

**Keywords:** Rhg1, SCN resistance, soybean, CNV, RNAseq, Fayette

## Abstract

Soybean yield loss due to soybean cyst nematode (SCN) infestation has a negative impact on the US economy. Most SCN-resistant soybeans carry a common resistance locus (*Rhg1*), but there is extensive copy number variation of a 31.2-kb segment at *Rhg1*. To identify the effects of *Rhg1* copy number on the plant prior to SCN infection, we investigated genome-wide expression profiles in isogenic Fayette plants carrying different copy numbers at the *Rhg1* locus (9–11 copies), that confer different levels of resistance to SCN. We found that even small differences in copy number lead to large changes in expression of downstream defense genes. The co-expression network constructed from differentially expressed genes (DEGs) outside the *Rhg1* locus revealed complex effects of *Rhg1* copy number on transcriptional regulation involving signal transduction and ethylene-mediated signaling pathways. Moreover, we report variation in expression levels of phytoalexin biosynthesis-related genes that is correlated with copy number, and the activation of different NBS-LRR gene sets, indicating a broad effect of copy number on defense responses. Using qRT-PCR time series during SCN infection, we validated the SCN responses of DEGs detected in the copy number comparison and showed a stable upregulation of genes related to phytoalexin biosynthesis in resistant Fayette lines during the early stages of the incompatible interaction between soybeans and SCN, before syncytium formation. These results suggest additional genes that could enhance *Rhg1*-mediated SCN resistance.

## Teaser

Soybean cyst nematode (SCN) infestation devastates soybean crops, impacting the US economy. The *Rhg1* gene, the most crucial for SCN resistance, exhibits varying copy number in soybeans. This study discovers significant changes in defense gene expression even with slight copy number variation. Chaiprom et al. also identified intricate regulatory pathways affected by *Rhg1* copy number. Additionally, during SCN infection, genes related to defense mechanisms, such as phytoalexin biosynthesis and NBS-LRR, showed heightened activity in resistant plants, offering insights into enhancing SCN resistance.

## Introduction

Soybean cyst nematode or SCN (*Heterodera glycines* Ichinohe) is a sedentary endoparasite that infects soybean roots, causing a dramatic decrease in soybean yield. SCN penetrates soybean roots as a second-stage juvenile (J2) and migrates intracellularly to establish a feeding cell near vascular tissues (Mitchum 2016). The nematode uses a stylet and secretes effector enzymes to destroy cell walls of neighboring cells to fuse them with the initial feeding cell and form a multinucleate cell, which is known as a syncytium (Acedo *et al*. 1984; Kim *et al*. 1987, 2010). During SCN infestation, the nematodes require the maintenance of a syncytium for its development until the reproductive stage (Mahalingam *et al*. 1998; Kim *et al*. 2012). In the susceptible response, the nematode successfully establishes and maintains the syncytium whereas syncytial necrosis and degeneration are observed in SCN-resistant lines as a resistant response (Kim *et al*. 1987).

The management of soybean cyst nematode (SCN) infestation in the field requires an integrated approach, combining the use of SCN-resistant soybeans, crop rotation, and seed treatments (Niblack 2005; Niblack *et al*. 2008). Genetic mapping studies in soybean germplasms have identified numerous QTL associated with resistance to SCN (Concibido *et al*. 1994, 2004; Webb *et al*. 1995; Guo *et al*. 2005, 2006; Kim *et al*. 2010; Cook *et al*. 2012, 2014; Bent 2022). Among the major QTL governing SCN resistance, *Rhg1* and *Rhg4* play critical roles. The *Rhg1* locus is particularly important, with copy number variation (CNV) of a 31.2 kb region identified as a key factor in conferring resistance (Cook *et al*. 2012). This region contains a repeat unit with four genes, three of which *Glyma.18G022400* (amino acid transporter), *Glyma.18G022500* (alpha-soluble NSF attachment protein or alpha-SNAP), and *Glyma.18G022700* (wound-inducible protein, WI12) are actively expressed in resistant lines (Cook *et al*. 2012, 2014). Resistance alleles at *Rhg1* vary, with the susceptible cultivar Williams 82 carrying a single copy of the repeat unit, while resistant cultivars Forrest (Peking-derived) and Fayette (PI 88788-derived) carry three and ten copies, respectively (Cook *et al*. 2014; Lee *et al*. 2015; Bayless *et al*. 2016). The Peking genotype belongs to a low-copy-number *Rhg1* group, whereas PI 88788 is categorized under the high-copy-number group (Cook *et al*. 2014). Further studies revealed that Fayette populations exhibit CNV ranging from 9 to 11 copies, correlating with varying levels of SCN resistance (Lee *et al*. 2016). The Fayette cultivar, developed by introgression of the *Rhg1* resistance locus from PI 88788 into the Williams background, was classified into the high-copy-number *Rhg1* group, where the locus contains two types of repeat units: multiple copies similar to PI 88788 (FA and FB) and a single copy akin to Williams 82 (W), collectively forming the *rhg1-b* allele (Cook *et al*. 2014; Lee *et al*. 2015). Additionally, the interaction between *Rhg1* and *Rhg4* is critical, especially in plants with the P-type *Rhg1* allele, where *Rhg4* is necessary to achieve full SCN resistance (Yu et al. 2016). Nonetheless, breeding for SCN resistance is challenged by the limited genetic diversity within cultivated soybean germplasm, which has driven breeders to explore wild relatives like *Glycine soja* for new sources of resistance (Kim and Diers 2013). Two notable QTL, *cqSCN-006* and *cqSCN-007*, derived from the wild soybean ancestor PI 468916, have been identified as potential novel sources for improving SCN resistance (Wang et al. 2001; Winter et al. 2007; Yu and Diers 2017). These findings highlight the importance of genetic variation and genomic structure at key QTL in determining the molecular mechanisms of SCN resistance.

Understanding the contrasting molecular mechanisms between susceptible and resistant responses of soybeans to SCN would be beneficial for the improvement of SCN resistance in soybeans, for example by improving understanding of how such resistance can be overcome by virulent nematode strains. Yeast two-hybrid interaction studies of the α-SNAP and *WI-12* genes repeated at the *Rhg1* locus (Dong *et al*. 2020; Dong and Hudson 2022) have indicated the involvement of the endomembrane and hormone systems in defense signaling from these loci. There have also been numerous gene expression studies to identify the candidate genes related to defense responses in PI 88788 (Klink *et al*. 2010, 2011; Matsye *et al*. 2011; Torabi *et al*. 2023). For *Rhg1*-mediated resistance, RNA microarray analysis comparing SCN-susceptible to SCN-resistant soybean near–isogenic lines (NILs) that differ at the *Rhg1* locus identified potential SCN-responsive genes such as genes encoding CC-NB-LRR proteins, hypersensitive-like response proteins, and salicylic acid signaling proteins (Kandoth *et al*. 2011). More recently, differential expression of early response genes was detected during the incompatible interaction between the resistant Fayette#99 line and SCN HG type 0 at 8 h post inoculation, during the migration phase (Miraeiz *et al*. 2020), suggesting that other defense-related genes may be involved in SCN resistance mediated by CNV at the *Rhg1* locus.

This study examined genome-wide gene expression profiles in soybean roots using RNAseq to identify differentially expressed genes (DEGs) between lines isolated from the same inbred population of the Fayette cultivar with variation in CNV at the *Rhg1* locus. A co-expression network was constructed to determine gene clusters and potential defense mechanisms involved in *Rhg1*-mediated SCN resistance. Candidate genes were selected based on their differential expression between the Fayette isolines and their previous identification as early response genes (Miraeiz *et al*. 2020). Then, we further validated dynamic expression of the candidate genes in soybean roots infected with SCN HG-type 0 at three time points (8, 24, and 48 h post inoculation), from migratory phase to the establishment of the syncytium stage, using high-throughput qRT-PCR.

## Materials and methods

### Plant materials and RNA extraction for RNAseq analysis of three Fayette lines

Three Fayette lines with CNV at the *rhg1-b* locus were selected from a homeolog-controlled (hc)TaqMan assay screen of a population of 102 Fayette plants (Lee *et al*. 2016). These 102 Fayette individuals were from an inbred, self-fertilized population of the Fayette cultivar (Bernard *et al*. 1988) that has been maintained by the department of Crop Sciences at the University of Illinois. Once isolated, these individuals were propagated as isolines by single seed descent. The three isolines used in this study were Fayette#01 (represents the lowest hcTaqMan fluorescence level in Fayette population predicted as the lowest copy number; low-copy Fayette), Fayette#19 (represents the intermediate fluorescence level in Fayette population; intermediate-copy Fayette), and Fayette#99 (represents the highest fluorescence level in Fayette population; high-copy Fayette). Using seeds harvested from a single plant, 10 vapor-sterilized seeds were germinated and grown in 10 cone-tainers (cone-shaped plastic pots) filled with an autoclaved mixture of sand and Turface (a packing clay used for sports turf) in a 2:1 ratio. The plants were maintained in the growth chamber for 15 days under 16/8 h light/dark condition at 26°C. For each Fayette line, whole roots were collected from four biological replicates and immediately flash-frozen in liquid nitrogen. Plant tissues were then kept at −80°C until RNA extraction. The frozen tissue was ground to fine powder with a mortar and pestle, homogenized in CTAB buffer prepared for RNA extraction (Chang *et al*. 1993) and purified in acidic phenol/chloroform. DNase I (New England Biolabs) was used to remove contaminated DNA according to manufacturer's protocol. The RNA was precipitated with isopropanol and resuspended in RNase-free water. Then, RNA concentration and quality were measured using a Nanodrop spectrophotometer (NanoDrop Technologies, Wilmington, DE, USA) and an Agilent Bioanalyzer. The RNA samples were sent for library preparation and sequencing to the DNA Services unit in the Roy J. Carver Biotechnology Center, University of Illinois at Urbana-Champaign, IL.

### RNAseq data preprocessing and differential expression analysis

The 100 nt paired-end reads were sequenced using the Illumina HiSeq2500 sequencing system at the Roy J. Carver Biotechnology Center. After quality check using FastQC (Andrews 2010), Trimmomatic (Bolger *et al*. 2014) was used to trim low quality bases (*q* < 20) from the ends of the reads, and any read that was left with fewer than 35 nucleotides were completely removed. Then, HiSat2 (Kim *et al*. 2015) was used to map high-quality reads against soybean (*G. max* cv. Williams 82) reference genome version Gmax_275_Wm82.a2.v1 (Schmutz *et al*. 2010), obtained from the Phytozome database (Goodstein *et al*. 2012). The reads that were mapped to exon regions were quantified for each gene, using featureCount (Liao *et al*. 2014). The identification of DEGs among three Fayette lines was performed by the ANOVA-like tests using the negative binomial generalized linear model implemented in edgeR (Robinson *et al*. 2010), which also adjusts for read depth and TMM normalization (Robinson and Oshlack 2010); the Benjamini–Hochberg false-discovery-rate adjusted *P*-value (FDR) cut-off was set at 0.05. The R Package ggplot2 (Gómez-Rubio 2017) was used for generating volcano plots and visualizing principal component analysis (PCA) of DEGs. The heatmap.2 function in the gplots (Warnes et al. 2022) R package was used to draw heatmaps of expression profiles and perform hierarchical clustering.

### Co-expression network analysis and clustering

The normalized expression values (transcripts per million) of DEGs were used to calculate the Pearson correlation coefficient for every pair of DEGs to create a square adjacency matrix. Using the R package igraph (Csardi and Nepusz 2006), the co-expression network was constructed based on strongly positively or negatively correlated co-expressed genes with the correlation coefficient cut-off set at ± 0.9. The network properties were examined to identify gene clusters, bridging genes that connect between two clusters, and hub genes. The Girvan–Newman algorithm (Newman and Girvan 2004) was applied to detect gene clusters based on edge betweenness. The edge betweenness represents the number of shortest paths that pass through an edge between gene pairs. High betweenness of an edge indicates that this edge tends to link between two genes from different clusters, while low edge betweenness is characteristic of a connection within the cluster. Similarly, the betweenness centrality of a node measures the number of shortest paths that pass through that node between two other nodes. The betweenness centrality score of a gene (i.e. a node) implies its topologically important location in the network whether the gene is a member of a cluster or a bridge between two clusters. In addition, the degree of connectivity was measured to identify highly connected genes or hub genes, which can be key regulators within gene clusters.

### Gene ontology (GO) term enrichment analysis and KEGG pathway annotation

The DEGs in each gene cluster in the co-expression network were identified and over-representation analysis performed on their assigned GO terms and Kyoto Encyclopedia of Genes and Genomes (KEGG) pathway assignments, compared to the annotations of all genes in the soybean genome as background. The GO term enrichment tool (Morales *et al*. 2013) embedded in the SoyBase website (Grant *et al*. 2010) was used to identify over-represented GO terms that were analyzed using Fisher's exact test and a Bonferroni correction for multiple testing, with cut-off set at *P* < 0.05. KEGG pathway annotation was performed using KOBAS (Xie *et al*. 2011) and BlastKOALA (Kanehisa *et al*. 2016).

### Transcription binding site analysis

A set of promoter sequences (1-kb sequences upstream from the transcript start site) of DEGs in each gene cluster was retrieved using the Biomart tool from the Phytozome website to examine enrichment analysis of transcription factor (TF) binding sites using the PlantTFDB 4.0 (Jin *et al*. 2017). The TFs and their motifs preidentified in the database were integrated from different sources such as literature, experiments, and computational prediction; however, the majority of these were derived from *Arabidopsis*, transferred to soybean through reciprocal best BLAST hits. The tool integrated two steps for the enrichment analysis. The first step is to scan for TF binding site motifs in promoter sequences based on find individual motif occurrences (Grant *et al*. 2011) with *P*-value cut-off at 10e-5. Then, Fisher's exact test was applied to identify over-represented motifs in the promoter sequence set with a higher frequency in the DEG cluster, compared to their frequency in the promoter sequences of all genes in the soybean genome, using an adjusted FDR *P*-value threshold of *P* < 0.01.

### Plant materials, nematode inoculation, and RNA extraction for qRT-PCR

We also compared this data to that described in the previous RNAseq analysis of soybean roots in response to SCN infection at 8 h post inoculation (Miraeiz *et al*. 2020) and performed qRT-PCR to validate and compare expression between the two experiments. For the lines from Miraeiz *et al*. (2020), soybean seeds were harvested from a single plant of each of four genotypes grown in greenhouse, including *Glycine max* cv. Williams 82, *G. max* cv. Peking, *G. max* cv. Fayette, and *G. soja* PI 468916. These different genotypes were selected to contrast different types of SCN resistance: Peking for P-type (Peking-derived, low-copy at *Rhg1* or *rhg1-a*), Fayette for F-type (PI 88788-derived, high-copy at *Rhg1* or *rhg1-b*), a wild soybean line PI 468916 with SCN resistance QTL *cqSCN-006* and *cqSCN-007*, and the susceptible control Williams 82 (wild type, single copy at *Rhg1*), the reference genotype, which is susceptible to SCN. The Fayette seeds for that experiment were obtained from a Fayette#99 plant harboring 11 copies of the 31.2-kb repeat segment at the *Rhg1* locus (Lee *et al*. 2016). The vapor-phase sterilized seeds were pregerminated at room temperature in the dark for three days to prepare for mock (water) and SCN inoculation. Before the inoculation, eggs of SCN HG type 0 were obtained from Alison Colgrove, University of Illinois at Urbana-Champaign. Utilizing the inoculum preparation described by Mahalingam *et al*. (1998) with some modifications, petri dishes containing eggs in distilled water were incubated at 27°C with constant gentle agitation of 50 rpm for 4 days. For inoculation, a 3-day-old seedling was placed on top of a sterilized germination paper (20 × 30 cm^2^), which was folded three times and moistened with distilled water in a 10-cm petri dish. Each seedling was inoculated by pipetting 1 mL of inoculum (1,500 J2 s) or 1 mL of distilled water onto the roots above the root tip and covered with a piece of moistened filter paper. The petri dishes were labeled, then placed randomly in plastic trays (50 × 30 × 10 cm) filled with ∼2 L of water, each tray covered with a humidity dome and double-layered with a semiclear plastic bag. The seedlings were maintained in the growth chamber under fluorescent lights of 16/8 h light/dark photoperiod at 26°C. Eight biological replicates of root tissues were harvested for each condition and flash frozen in liquid nitrogen at three time points, including 8, 24, and 48 h post inoculation (hpi). Root samples designated for 24 and 48 hpi time points were rinsed at 8 hpi to prevent nematodes from further entering. All tissue samples were kept in a −80°C freezer until RNA extraction. Total RNA was extracted from individual root samples using E-Z® 96 Plant RNA Kit (Omega Bio-Tek) and treated with DNase I (Thermo Scientific) according to manufacturer's protocol. The RNA quality was assessed with a NanoDrop ND-1000 spectrophotometer (NanoDrop, Wilmington, DE, USA) and 1% agarose gel electrophoresis.

### Quantitative real-time RT-PCR (qRT-PCR)

The total RNA was used for cDNA synthesis using the High-Capacity cDNA Reverse Transcription Kit (Applied Biosystems). This included four biological replicates of cDNA samples for 8 and 24 hpi, and three biological replicates for 48 hpi. Primer sets were designed for the target genes using NCBI Primer-Blast tool (Ye *et al*. 2012). Then, cDNA samples and primer sets were prepared to perform qPCR at the Functional Genomics Unit of Roy J. Carver Biotechnology Center, University of Illinois at Urbana-Champaign, IL. The samples were processed on the Fluidigm Dynamic Array integrated fluidic circuits for gene expression and the BioMark^TM^ HD system (Fluidigm Corporation, USA) according to the manufacturer's procedures (Real-Time PCR Analysis User Guide, PN 68000088). For differential expression analysis, Ct values were obtained from the Biomark Real-time PCR Software (version 4.3.1) and normalized by geometric mean of three reference genes, including *Glyma.12G051100* (*SKIP16*), *Glyma.20G130700* (*TIP41*), and *Glyma.20G141600* (Ubiquitin). Using the R package limma (Ritchie *et al*. 2015), log_2_ relative expression values of each gene normalized by internal gene references were used to fit a linear model, followed by estimation of variances using the empirical Bayes approach. For each gene and genotype, log_2_ fold change values were calculated from pair-wise comparisons between SCN-infected vs mock-inoculated roots. The DEGs were identified at an adjusted *P*-values cut-off of 0.05. The heat map showing expression profiles was drawn using the R Package ggplot2 (Gómez-Rubio 2017).

## Results

### Differentially expressed genes from RNAseq analysis of three Fayette lines

In total, 465 DEGs were identified between Fayette lines with CNV at the *Rhg1* locus, including Fayette#01 (low-copy Fayette), Fayette#19 (intermediate-copy Fayette), and Fayette#99 (high-copy Fayette) (Supplementary Fig. 1a). The PCA revealed a clear separation between the three Fayette isolines (Supplementary Fig. 1b). The hierarchical clustering showed that the expression of DEGs in Fayette#19 was more similar to those in Fayette#99 than Fayette#01 (Supplementary Fig. 1c). Expression level differences at the three *Rhg1* genes between these Fayette lines did not reach statistical significance. In comparison to our previous RNAseq study (Miraeiz *et al*. 2020), 58 of 465 DEGs responding to CNV in Fayette lines were found in common with DEGs between the lines in soybean roots infected with SCN HG-type 0 vs mock inoculated with water at 8 h post inoculation (hpi) (Supplementary Fig. 2). Considering the response to SCN infection among four soybean lines, statistically significant changes in expression of 15 genes from 58 overlapped DEGs between experiments were identified uniquely in the resistant Fayette#99 isoline, or in common between Fayette#99 and non-Fayette genotypes (resistant Peking, resistant *G. soja* PI 468916, and susceptible Williams 82) in response to migrating SCN. The other 43 early response genes were differentially expressed only in non-Fayette genotypes. These four genotypes shared induction of four genes (*Glyma.09G049200*, *Glyma.11G062500*, *Glyma.11G062600*, and *Glyma.16G195600*) in SCN-infected roots. Interestingly, in this study, these genes were more highly expressed in Fayette#99 compared to the other, lower copy Fayette lines. This overlap in DEGs suggested that some genes expressed differentially between Fayette lines due to CNV at the *Rhg1* locus are likely also involved in the *Rhg1*-mediated resistance to SCN at the early time points (Supplementary Fig. 2).

### Co-expression network of DEGs in response to CNV at Rhg1

**Fig. 1.**
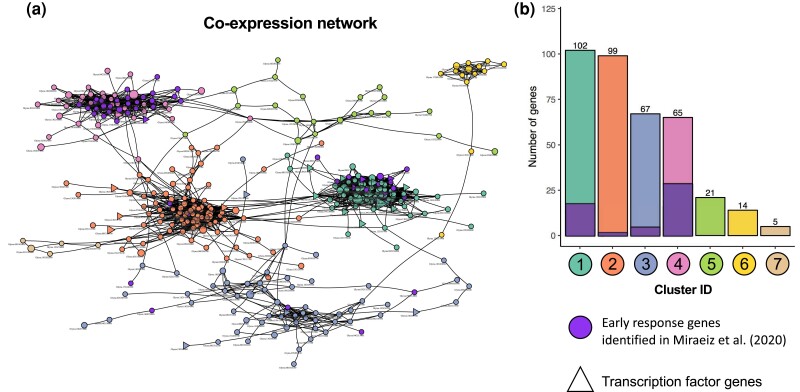
Co-expression network of DEGs and gene clusters. a) A co-expression network constructed from strongly co-expressed DEGs with the Pearson correlation coefficient cut-off set at ±0.9. Colors represent gene cluster membership (see panel b), and genes previously identified as SCN early response genes (Miraeiz *et al*. 2020), triangles indicate annotated TF-encoding genes. b) Bar chart showing number of DEGs and early response genes in each of seven clusters identified based on edge betweenness using Girvan–Newman algorithm.

The expression levels of 465 DEGs across all 12 samples of Fayette lines were normalized, and the Pearson correlation coefficient of every gene pair calculated. After removing weakly correlated genes (|*r*| < 0.9), a co-expression network was constructed from 393 strongly correlated DEGs (3,731 interactions), consisting of 10 network components. The largest component was made up of 373 co-expressed DEGs and 3,720 interactions (Fig. 1a) while the other nine components consisted of either two or three co-expressed genes. For the largest subnetwork, the degree of connectivity ranged from one to 72 with a mean of 19.95. The gene clusters identified based on edge betweenness in this subnetwork accounted for seven clusters (clusters 1–7), consisting of 102, 99, 67, 65, 21, 14 and five gene members in each cluster, respectively (Fig. 1b). In addition, 54 early response genes were clustered in only the first four clusters; however, the majority of these genes belonged to either cluster 1 (18 genes) or cluster 4 (29 genes).

### Expression profiles of DEGs in each network cluster and their predicted functions

**Fig. 2.**
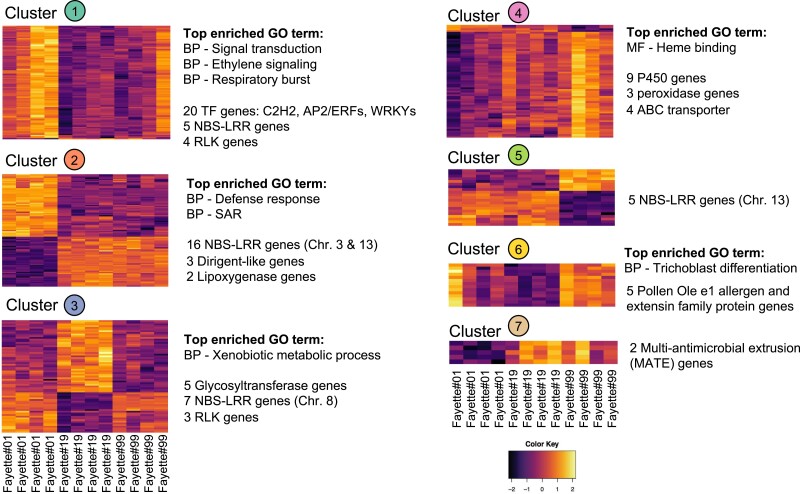
Heatmap showing gene expression profiles and over-represented GO terms in each cluster. The clusters are described in Fig. 1. The colors in the heatmap represent normalized relative changes in expression levels in each sample. Each row in the heatmaps represents the genes forming the associated cluster. Heatmaps are arranged from cluster 1 at the top left through cluster 7 at the bottom right. BP stands for enriched GO terms in the biological process category. MF stands for enriched GO terms in molecular function category.

The heatmap and GO term enrichment analysis revealed distinct expression profiles of DEGs and their functions in each cluster (Fig. 2). The gene clusters were detected based on the edge betweenness in a co-expression network; thus, each cluster contained genes that showed both up- and down-regulation in specific group of samples, interpreted as representing a group of genes involved in related biological responses that were activated in specific samples.

**Fig. 3.**
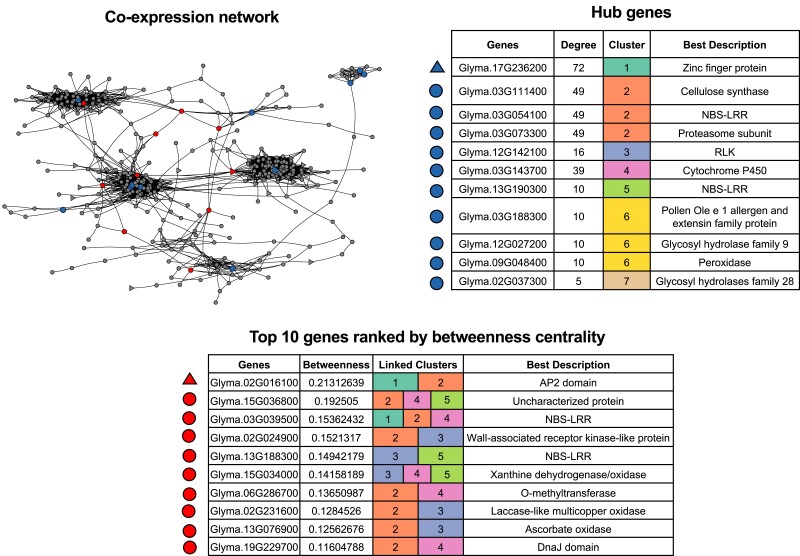
Hub genes in each cluster and top 10 genes ranked by betweenness centrality. The network diagram at top left shows *“*hub” genes identified in the network analysis are shown in blue (gene details at top right) and the top 10 genes with highest betweenness centrality are shown in red (gene details at bottom). Triangles indicate annotated TF-encoding genes.

The network properties of bridging genes, that connect between two clusters, and hub genes were examined in each cluster (Fig. 3). The largest cluster (cluster 1) represented a set of genes that were highly expressed in Fayette#01. A gene encoding a C2H2 zinc finger transcription factor (*Glyma.17G236200*) had the highest degree of connectivity (72 neighbors). Moreover, a total of 21 transcription factor (TF) genes were found in this cluster, including seven APETALA2/Ethylene responsive factor (AP2/ERF) genes, five WRKY genes (*Glyma.05G215900*, *Glyma.08G021900*, *Glyma.09G061900*, *Glyma.16G026400*, and *Glyma.19G254800*), four C2H2 zinc finger genes (*Glyma.04G044900*, *Glyma.06G045400*, *Glyma.17G236200*, and *Glyma.20G133200*), two C3H zinc-finger genes (*Glyma.02G296600* and *Glyma.14G016300*), and one gene of each three other TF families (bZIP, NAC, and HSF). Interestingly, the GO term enrichment analysis showed 11 over-represented terms in this cluster such as intercellular signal transduction (GO:0035556), ethylene-mediated signaling pathway and biosynthetic process (GO:0009873 and GO:0009693) and respiratory burst involved in defense response (GO:0002679) (Fig. 2). Further investigation showed that some genes were classified with several GO terms; therefore, this cluster represented a group of genes involved in multiple biological processes related to defense signaling.

Cluster 2 contained the three highest connectivity genes (*Glyma.03G054100,* encoding a nucleotide-binding site-leucine-rich repeat (NBS-LRR), *Glyma.03G073300*, encoding a proteasome subunit alpha type-2-A, and *Glyma.03G111400*, encoding a cellulose synthase-like protein). These genes were strongly correlated to each other, and directly connected to 49 other genes. The cluster represented a group of defense-related genes (GO:0006952) such as 16 NBS-LRR genes (mostly located in complex NBS-LRR loci on chromosome 3 and 13), three dirigent-like genes (*Glyma.03G044300*, *Glyma.03G045600*, and *Glyma.03G046000*) and two lipoxygenase genes (*Glyma.04G105900* and *Glyma.15G026500*). Unexpectedly, approximately half of the NBS-LRR genes in cluster 2 were found in close genomic proximity with one another, on chromosome 3. These genes are closely related to the *NBS-LRR Rps-k-2* gene (Resistance to *Phytophthora sojae*) identified in soybean (Gao and Bhattacharyya 2008). In addition, the heatmap showed that the defense-related genes in this cluster were either intensely up-regulated or down-regulated in Fayette#01, compared to those in the other two lines (Fig. 2). Moreover, similar expression patterns were found in cluster 7, the smallest cluster. Two genes out of five total genes in cluster 7 encode transporter proteins in the multiantimicrobial extrusion family.

In cluster 3, genes were significantly induced or suppressed in Fayette#19, compared to those in Fayette#01 and Fayette#99 (Fig. 2). The gene with the highest degree of connectivity was a receptor-like kinase (RLK) gene (*Glyma.12G142100*), connected to 16 other genes. The over-represented GO terms were related to xenobiotic catabolic and metabolic process (GO:0042178 and GO:0006805), including five glycosyltransferase genes (*Glyma.08G338500*, *Glyma.08G338600*, *Glyma.08G338700*, *Glyma.08G338900*, and *Glyma.15G054500*) (Fig. 2). Additionally, two other RLK genes (*Glyma.06G261000* and *Glyma.09G240700*) and seven NBS-LRR genes were also found in this cluster. Most of these NBS-LRR genes were again found in proximity, in complex NBS-LRR loci on chromosome 8 (*Glyma.08G317400*, *Glyma.08G317700*, *Glyma.08G318000*, *Glyma.08G319300*, and *Glyma.08G323200*) and their sequences had similarity to the *RPM1* genes encoding NSB-LRR disease resistance to *Pseudomonas syringae* in *Arabidopsis* (Grant *et al*. 1995).

Cluster 4 had 16 of 65 total genes related to oxidation-reduction process (GO:055144). This GO term was not significantly enriched in the biological process category; however, 12 of these genes were also classified in heme binding (GO:0020037), which was one of the significantly over-represented GO terms in the molecular function category, including nine cytochrome P450 (P450) genes and three peroxidase genes (*Glyma.01G130800*, *Glyma.03G038200*, and *Glyma.03G038700*) (Fig. 2). One of these P450 genes, *Glyma.03G143700*, was found to be the hub gene, with its expression showing a strong correlation with 39 other genes. The expression levels of genes in this cluster tended to correlate with the CNV at the *Rhg1* locus, with genes being highly expressed in Fayette#99 (high copy), less expressed in Fayette#19 (intermediate copy), and least expressed in Fayette#01 (low copy) (See cluster 7 in Fig. 2).

The most frequent gene annotation in cluster 5 is the NBS-LRR family, and these genes lie tightly together on chromosome 13. The predicted closest protein homologs of these genes were NBS-LRR RPG1-B, conferring resistance to *P. syringae* in soybean (Ashfield *et al*. 2004). One of these was a hub gene (*Glyma.13G190300*) connected to 10 genes in the cluster. The heatmap showed up-regulation or down-regulation of genes in Fayette#99 relative to those in two other Fayette lines (Fig. 2). Similarly, Fayette#99 showed high expression levels of genes in cluster 6 in comparison to those in other lines (Fig. 2). This cluster represented a group of genes related to trichoblast differentiation (GO:0010054) such as five genes annotated as “pollen Ole e1 allergen” and extensin family proteins (*Glyma.03G188300*, *Glyma.10G016600*, *Glyma.11G232100*, *Glyma.18G025200*, and *Glyma.19G188400*).

### Bridging between gene clusters

The top 10 genes ranked by betweenness centrality, linking the clusters in the co-expression network, had diverse functional annotations (Fig. 3). Of these, eight genes acted as connectors between cluster 2 and other clusters. The gene with the highest betweenness in this network was an AP2/ERF TF gene (*Glyma.02G016100*), bridging between clusters 1 and 2. AP2/ERF is a TF superfamily that plays regulatory roles in plant development and stress responses. This gene was directly connected to different types of genes, of particular interest was *Glyma.11G228100* encoding a homolog protein of HSPRO, an Arabidopsis ortholog of a beet cyst nematode resistance gene (Cai *et al*. 1997); we also observed RLK genes (*Glyma.08G255000* and *Glyma.12G002400*), and NBS-LRR genes (*Glyma.03G039500*, *Glyma.06G259400* and *Glyma.13G194100*). Interestingly, one of these NBS-LRR genes (*Glyma.03G039500*) was connecting three clusters (clusters 1, 2, and 4). Another NBS-LRR gene (*Glyma.13G188300*) was a connector of two clusters (clusters 3 and 5). In addition, cell wall-related genes are included in both clusters 2 and 3, such as *Glyma.02G024900* encoding a wall-associated receptor kinase, *Glyma.02G231600* encoding laccase gene, and *Glyma.13G076900* encoding an l-ascorbate oxidase gene.

### The enrichment analysis of TF motif binding sites

**Fig. 4.**
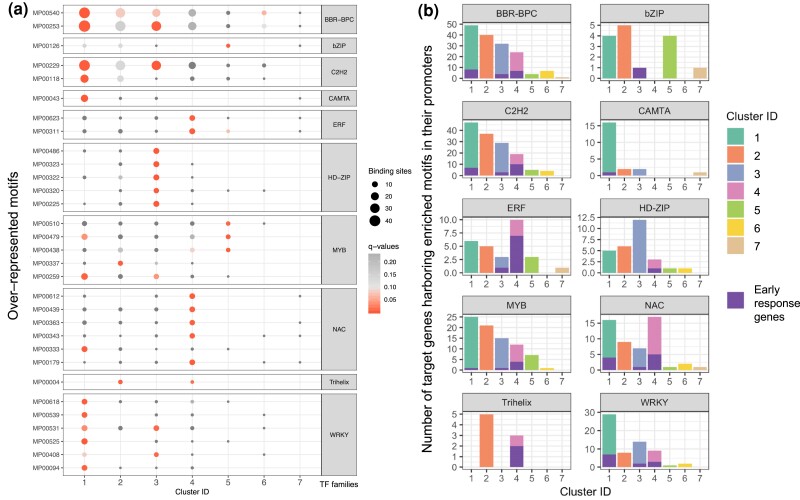
Enrichment analysis of TF binding motifs and number of genes harboring over-represented motifs in their promoters. a) A total of 31 over-represented motifs were identified in the promoters of DEGs in this study, by comparing motifs found in promoter sequences of DEGs in each cluster to the proportion of genes with one or more motif in the promoters of all genes in the soybean genome. The over-represented motifs were classified into those representing binding sites of 10 TF families. The size of the circle represents the number of genes harboring each motif. The colors represent adjusted *P*-values (Benjamini–Hochberg) or *q*-values. The significantly over-represented motifs were identified using Fisher's exact test, with FDR-adjusted *P*-value threshold < 0.01. b) Bar charts show the number of unique genes harboring over-represented motifs in their promoters. The motifs were classified into 10 TF families: BBR-BPC, bZIP, C2H2, CAMTA, ERF, HD-ZIP, MYB, NAC, Trihelix, and WRKY. The colors of the upper bar represent the seven gene clusters by expression, the purple color in the lower bar represents the fraction of early response genes that were both differentially expressed in response to SCN infection at 8 h post inoculation (hpi) based on previous RNAseq analysis (Miraeiz *et al*. 2020) and differentially expressed in response to *Rhg1* copy number.

The statistical test for enrichment of regulatory motifs in the promoter sequences (1-kb sequences upstream from the transcript start site) of DEGs was examined in each gene cluster, using the Fisher exact test with the adjusted *P*-value (Benjamini–Hochberg) or q value cut-off set at 0.01, compared to the frequency of those sites found in the promoter sequences of all genes in the soybean genome as background. In total, 31 over-represented binding-site motifs were identified and categorized into 10 TF families predicted to bind them, including six WRKY (single-letter amino acid code) motifs, six NAC (NAM, ATAF, and CUC) motifs, six HD-ZIP (homeodomain-leucine zipper) motifs, five MYB (myeloblastosis) motifs, two C2H2 (Cys2-His2) motifs, two ERF (ethylene response factor) motifs, two BBR-BPC (barley B recombinant/basic pentacysteine) motifs, and one motif of each of bZIP (basic leucine zipper), Trihelix, and CAMTA (calmodulin-binding transcription activator) (Fig. 4a, Supplementary Fig. 3). Of seven gene clusters, the significantly over-represented motifs were detected in the promoter sequences of only five clusters (clusters 1, 2, 3, 4, and 5). The binding motifs of five TF families (BBR-BPC, C2H2, WRKY, MYB, and NAC) were significantly over-represented in multiple clusters (Fig. 4a). For example, three TF families (BBR-BPC, C2H2, and WRKY) were predicted to regulate genes in clusters 1 and 3, whereas MYB motif binding sites were significantly enriched in the promoters of DEGs in three clusters (clusters 1, 2, and 5). We also observed cluster-specific motifs: the DEGs in cluster 1 were targeted by one CAMTA motif. The sites of six HD-ZIP motifs were over-represented in the promoters of DEGs in cluster 3. Moreover, enriched recognition motifs of the ERF family were identified in the promoters of DEGs in cluster 4 (Fig. 4a). The binding motif of the bZIP TF family was enriched in cluster 5, and a Trihelix motif was significantly over-represented among 99 DEGs in cluster 2. Considering all target genes of each TF family (Fig. 4b), the binding sites of major TF families (e.g. BBR-BPC, C2H2, and MYB) were generally found in the promoters of DEGs in numbers correlated with the number of DEGs in each cluster. However, DEGs with some TF family binding sites in their promoters one or more times tended to be over-represented in specific clusters, regardless of the total number of DEGs in each cluster, for example, the target DEGs of ERF and NAC TF families in cluster 4 outnumbered those in other clusters.

### Expression profiling of genes in response to SCN at 8, 24, and 48 hpi by qRT-PCR

**Fig. 5.**
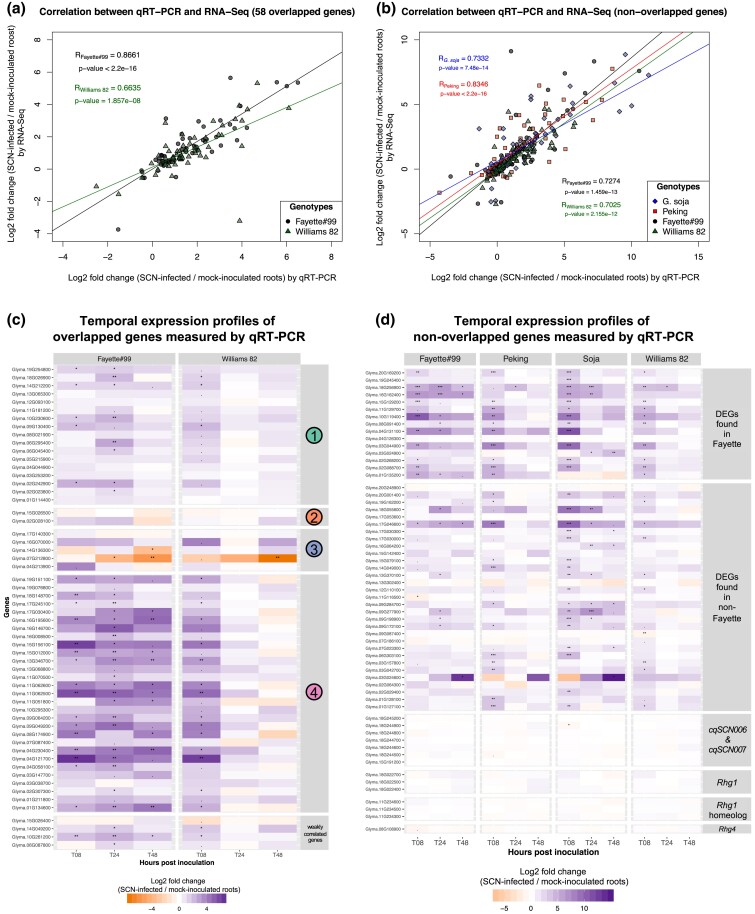
Temporal expression profiles of candidate genes measured by qRT-PCR using the fluidigm biomark HD system. The log_2_ fold change value represents the ratio between the average normalized expression level of a gene in SCN–infected roots to that in mock-inoculated roots. a) Scatter plot shows correlation between log_2_ fold change values of overlapped genes measured by RNAseq (Miraeiz *et al*. 2020), x axis, and qRT-PCR, y axis, at 8 hpi in Fayette#99 (black circle) and Williams 82 (green triangle). b) Scatter plot shows correlation between log_2_ fold change values of nonoverlapped genes measured by RNAseq (Miraeiz *et al*. 2020) and qRT-PCR at 8 hpi in Fayette#99 (black circle), Peking (red square) and *G. soja* PI 468916 (blue diamond) and Williams 82 (green triangle). c) Heatmap shows expression profiles of overlapped genes at 8, 24, and 48 hpi in Fayette#99 and Williams 82. Overlapped genes are those that were differentially expressed in early response to SCN infection at 8 h post inoculation (hpi) and also differentially expressed in response to *Rhg1* copy number. The genes were grouped by the gene clusters identified in the co-expression network. d) Heatmap shows expression profiles of nonoverlapped genes at 8, 24, and 48 hpi in Fayette#99, Peking, *G. soja* PI 468916 and Williams 82. Nonoverlapped genes denote genes that were differentially expressed in early response to SCN infection at 8 hpi, but not differentially expressed in response to *Rhg1* copy number. The genes were grouped into two main categories: 1) DEGs previously identified in RNAseq analysis (Miraeiz *et al*. 2020) including DEGs found in Fayette and DEGs found in non-Fayette genotypes. 2) All genes (not just DEGs) located in SCN resistance QTL including *cqSCN006* and *cqSCN007*, *Rhg1*, *Rhg1* homolog, and *Rhg4*. The colors in the heatmap represent up-regulation (purple) and down-regulation (orange). The statistically significant changes in gene expression were identified using an FDR-adjusted *P*-value cut-off set at 0.05. (0–0.001 “***,” 0.001–0.01 “**,” 0.01–0.05 “*,” and 0.05–0.1 “.”).

Primer sets were designed for 121 selected genes (Supplementary Table 1) in order to validate the RNAseq analysis results on Fayette lines with CNV at *Rhg1*, and our previous study in early response to SCN infection (Miraeiz *et al*. 2020). This included 58 DEGs in response to CNV in the three Fayette lines which overlapped with early response genes induced by the SCN infection at 8 hpi (overlapped genes), 46 other selected early-response genes in SCN-infected plants which were not significantly differentially expressed in response to CNV at *Rhg1* (nonoverlapped genes), 14 candidate genes located in major SCN-resistance QTL (*Rhg1*, *Rhg4*, *cqSCN-006*, and *cqSCN-007*), and three reference genes (Supplementary Fig. 2). For statistical analysis, the DEGs were identified between SCN-infected roots vs mock-inoculated roots of each genotype and each time point using the R package limma with the FDR-adjusted *P*-value cut-off set at 0.05. For overlapped genes, the log_2_ fold change values (SCN-infected/mock-inoculated roots) estimated by qRT-PCR were closely correlated with those from the previous RNAseq data in Miraeiz *et al*. (2020) (R_Fayette#99_ = 0.8661 and R_Williams 82_ = 0.6635), validating the pattern of expression observed in the current RNAseq experiment (Fig. 5a). We attribute the differences to biological variations in the plant-nematode experimental system, the lower signal/noise in the Williams comparison, the variability in RNAseq and qPCR workflows, and the different statistical analyses employed. The qRT-PCR results showed statistically significant changes in expression levels of 36 out of 58 overlapped genes in response to SCN invasion in at least one genotype (Fig. 5c). The majority of genes were transiently induced at 8 hpi in susceptible Williams 82, but those remained up-regulated until 24 or 48 hpi in resistant Fayette#99, especially overlapped genes in cluster 4. For example, these transiently induced genes included four cytochrome P450 genes (*Glyma.09G049200*, *Glyma.11G062500*, *Glyma.11G062600*, and *Glyma.15G156100*), endochitinase PR-4 (*Glyma.13G346700*), Glycinol-4-dimethylally transferase (*Glyma.01G134600*), a gene encoding stress-induced protein SAM22 (*Glyma.17G030400*), a gene encoding ATP-binding cassette transporter protein (*Glyma.15G012000*), and a bHLH TF gene (*Glyma.09G064200*).

The temporal expression profiles were further investigated for nonoverlapping genes that were altered during SCN infection at 8 hpi in either Fayette or non-Fayette genotypes. A strong correlation between the qRT-PCR and previous RNAseq data were found in all four genotypes (R_Fayette#99_ = 0.7272, R_Williams82_ = 0.7025, R_Peking_ = 0.8346, R*_G. soja_* = 0.7332) (Fig. 5b). As expected, these nonoverlapping genes were induced at 8 hpi and predominantly found in *G. soja,* which had a wide range of gene expression changes in response to SCN infection, validating the previous RNAseq data (Fig. 5d). The qRT-PCR results showed 38 DEGs in at least one genotype. Of these, 18 DEGs were transiently induced at 8 hpi in both susceptible and resistant genotypes, for example, a RLK gene (*Glyma.02G088700*), a calmodulin gene (*Glyma.03G157800*), an ACC-oxidase gene (*Glyma.02G268200*), two dirigent-like genes (G*lyma.01G127100* and *Glyma.03G044900*), two peroxidase genes (*Glyma.15G129200* and *Glyma.20G169200*), and a beta-glucosidase gene (*Glyma.11G129700*). Moreover, some genes were continuously up-regulated in resistant lines at 8 hpi and later time points, for example, the P450 gene CYP82A (*Glyma.01G135200*), a predicted eugenol synthase gene (*Glyma.04G131100*), a gene encoding a subtilisin-like serine endopeptidase family protein (*Glyma.10G119400*), a tryptophan aminotransferase-related gene (*Glyma.16G162400*), a gene related to flavin-dependent monooxygenase encoding genes (*Glyma.17G046600*), and a gene predicted to encode a quinohemoprotein ethanol dehydrogenase (*Glyma.18G256900*). Interestingly, other genes were induced at two or three time points only in *G. soja*, including three peroxidase genes (*Glyma.09G277900*, *Glyma.09G284700*, and *Glyma.18G055600*), two WRKY genes (*Glyma.07G023300* and *Glyma.13G370100*), one chitinase gene (*Glyma.03G024900*) and one LRR-RLK (*Glyma.16G064200*). The significant down-regulation of a gene encoding a likely inositol transporter (*Glyma.09G087400*) was uniquely found in SCN-infected roots of susceptible Williams 82 at 8 hpi and was perfectly confirmed by the RNAseq experiment.

In addition, the expression of genes located in major SCN-resistance QTL intervals (*Rhg1*, *Rhg4*, *cqSCN-006*, and *cqSCN-007*) was measured during the SCN infection. A significant change in expression was not detected for any except for *Glyma.18G244900* in the *cqSCN-007* QTL interval (Fig. 5d). However, a *p*-nitrophenyl phosphatase (*Glyma.18G244900*) within the cqSCN-007 interval on chromosome 18 was significantly down-regulated at 8 hpi in *G. soja*.

## Discussion

### Isogenic Fayette lines are a useful tool to investigate signaling in Rhg1 SCN resistance

A gene expression study in the SCN syncytium was previously performed on resistant and susceptible soybean NILs differing at the *Rhg1* locus developed from *G. max* cv. Evans and PI 209322 (Kandoth *et al*. 2011). However, such NILs still contain many genetic differences. In this study, we investigated impacts of CNV at the *Rhg1* locus on genome-wide transcriptome changes between lines derived from the Fayette cultivar, which emerged in an inbred, self-fertilized population. Therefore, we report putative defense mechanisms involved in the SCN resistance conferred by relatively small changes in the CNV at the *Rhg1* locus, focusing on a high-copy number *Rhg1-*type SCN-resistant cultivar derived from PI 88788, a major resistance source. Using these isolines of the Fayette cultivar for this RNAseq analysis reduced noise from variation in genetic background relative to the previously investigated NILs, and shows that these isolines are largely isogenic. The hcTaqMan assay screen (Lee *et al*. 2016) revealed a wide distribution of copy number at the *Rhg1* locus within isolines of the Fayette cultivar. Both the low-copy Fayette#01 and the intermediate-copy Fayette#19 were predicted to most likely have 9 copies, yet with higher signal from #19, while the high-copy-Fayette#99 likely harbors 11 copies, determined by the significant differences in fluorescence signals compared with 9 and 10 copy controls. In addition, the high-copy-Fayette#99 showed a higher degree of resistance to SCN HG Type 2.5.7 than the intermediate-copy-Fayette#19 based on the SCN bioassay experiment (Lee *et al*. 2016). To investigate transcriptomic variation, we used RNAseq analysis to show 465 genes differentially expressed between replicates of whole roots in Fayette lines with CNV at the *Rhg1* locus, suggesting a complex signaling pathway in *Rhg1*-mediated SCN resistance that is dependent on copy number. The hierarchical clustering showed higher degrees of similarity in gene expression patterns between the intermediate-copy-Fayette#19 and the high-copy-Fayette#99 than those in the low-copy-Fayette#01, pointing out possible common mechanisms of gene regulation among Fayette lines with higher copy number at the *Rhg1* locus that need to be further explored (Supplementary Fig. 1c). Importantly, the RNAseq and qRT-PCR results showed that the significant changes in expression of the three *Rhg1* genes themselves were not detected in whole roots in Fayette lines with CNV at *Rhg1* at 15 days after planting, indicating that the underlying differences in *Rhg1* expression are presumably subtle and transient in this condition. As the differences in *Rhg1* copy number among Fayette isolines (9 through 11) would be anticipated to create differences in gene expression in the 10%–20% range, these would likely require substantially higher resolution than available from RNAseq or qRT-PCR to be detected. However, the correlation between CNV and gene expression of DEGs observed among these Fayette isolines suggests that, despite the predicted small changes in expression of the genes at the *Rhg1* locus, the impact of this variation is both nonlinear and substantial. Moreover, the *three Rhg1* genes were not significantly altered in whole roots of resistant Fayette#99 infected with SCN at 8, 24, and 48 hpi, suggesting transcript levels of *Rhg1* genes in Fayette lines are relatively stable. Matsye *et al*. (2011) previously identified the expression of two *Rhg1* genes, an amino acid transporter (*Glyma.18G022400*) and alpha-SNAP (*Glyma.18G022500*), in the syncytia of SCN infected roots at three time points (3, 6, and 9 days postinoculation) in both Peking and PI 88788. In addition, high expression of genes encoding dysfunctional *Rhg1*-resistance-type alpha-SNAPs, which were highly accumulated in the feeding cells, are thought to induce cytotoxicity to support the degeneration of the syncytium (Bayless *et al*. 2016). In terms of protein levels, the coevolution of defective forms of *Rhg1*-resistance-type alpha-SNAPs and its partner proteins (*N*-ethylemaleimide sensitive factor, NSF_RAN07_) was found to be a key mechanism in vesicle trafficking to balance between plant viability and cytotoxicity for the *Rhg1*-mediated resistance (Bayless *et al*. 2018). The characterization of alpha-SNAPs indicated that the expression of the *Rhg1* genes may differ extensively and locally in the syncytia. The atypical *Rhg1*-resistance-type alpha-SNAP proteins thus provide both benefits and drawbacks for plants. We speculate that the expression levels of *Rhg1* genes in Fayette lines may be precisely controlled to maintain steady expression in most tissues of the plant, but with possible strong differential expression in the syncytium cell itself after infection.

### Co-expression network of DEGs revealed key regulators and uncovered a complex regulatory network involved in SCN resistance

More than half of the DEGs in the co-expression network were expressed at higher or lower levels in the Fayette#01 line (low-copy Fayette) than those in other Fayette lines, clustering in the two largest clusters (clusters 1 and 2) and the smallest cluster (cluster 7). The majority of the genes encoding TFs were clustered in cluster 1, including genes encoding TFs in the C2H2 zinc finger, WRKY and AP2/ERF families. These TF families are classified in multiple over-represented GO terms, which are related to signal transduction, hormone signaling, and respiratory bursts, suggesting that the CNV at the *Rhg1* locus may confer SCN resistance via many TFs mediating transcriptional regulation in several biological processes. More similar expression patterns of these genes were found in the intermediate-copy-Fayette#19 and the high-copy-Fayette#99, which tend to cluster together. These two Fayette lines may thus share more transcriptional mechanistic similarity during SCN infection to each other than to the low-copy-Fayette#01. Based on sequence homology, a total of four genes (*Glyma.04G044900*, *Glyma.06G045400*, *Glyma.17G236200*, and *Glyma.20G133200*) in cluster 1 were homologous to the SALT TOLERANCE ZINC FINGER (STZ/ZAT10) encoding gene in *Arabidopsis*. One of these (*Glyma.17G236200*) was a hub gene closely related to a gene encoding the soybean cold-inducible zinc finger (SCOF-1) protein (Kim *et al*. 2001). The functional characterization of *SCOF-1* in soybean and *ZAT10* in *Arabidopsis* suggested that these genes regulate plant defense responses to abiotic stresses (Kim *et al*. 2001; Mittler *et al*. 2006), enhance tolerance to photoinhibitory light and exogenous H_2_O_2_ (Rossel *et al*. 2007), and are involved in early responsive suppression of jasmonate biosynthesis-related genes (Pauwels and Goossens 2008). In addition, Yuan *et al*. (2018) found that this set of C2H2 zinc finger genes plays a role in legume-rhizobia symbiosis and could form a complex transcriptional regulatory network by interacting with genes encoding WRKYs and several protein kinases such as MAPK, cGMP-dependent protein kinases and 5′AMP-activated protein kinases. In this study, the qRT-PCR results illustrated that several C2H2 zinc finger genes (*Glyma.04G044900* and *Glyma.06G045400*) and a WRKY gene (*Glyma.19G254800*) were induced in SCN infected roots in resistant genotypes, suggesting that these genes perhaps control plant defense responses to biotic stress, including SCN infection. Interactions of the C2H2 genes with other TFs and genes in signaling pathways were predicted in the co-expression network, indicating a complex transcriptional regulatory network involved in *Rhg1*-mediated SCN-resistance.

### Activation of different sets of R genes may lead to diverse defense mechanisms involved in Rhg1-mediated SCN resistance

As the largest group of resistance genes, NBS-LRR genes encoding intercellular immune receptors mediate effector-triggered immunity in response to pathogen attack. In the co-expression network we developed, NBS-LRR genes were identified in several gene clusters (e.g. clusters 1, 2, 3, and 5) and are also categorized into “defense response,” one of the over-represented GO terms in cluster 2. These included two hub genes and two bridging genes in the network. Two possible interpretations of this are that the SCN resistance conferred by the CNV at the *Rhg1* locus (1) involves NBS-LRR genes functioning as main players in detecting SCN and activating downstream defense mechanisms, and/or (2) is a multipurpose signaling system that also activates receptors for multiple pathogens. Most of the NBS-LRR genes in plant genomes are organized in genomic clusters. The differentially expressed NBS-LRR genes in these Fayette lines showed distinct expression patterns in each network cluster and high correlation with other NBS-LRR genes in proximity within complex NBS-LRR gene cluster loci (mostly on chromosome 3, 8, and 13). These genes were homologous to well-characterized resistance genes and located within QTL for resistance to other diseases. For example, the NBS-LRR genes in cluster 5, which showed distinct expression levels in Fayette#99 from those in other lines, were closely related to the RPG1-B resistance gene for *P. syringae* in soybean (Ashfield *et al*. 2004). RPG1-B indirectly detects pathogen effectors by interacting with RIN4-like proteins in soybean (Selote and Kachroo 2010), indicating that these differentially expressed NBS-LRR genes in Fayette lines perhaps act as guards and recognize a broad spectrum of effectors. Based on the observations in isogenic Fayette lines with CNV at *Rhg1*, these sets of NBS-LRR genes were predicted to interact with different types of defense-related genes within and between network clusters. Thus, we hypothesize that an alteration of expression in these NBS-LRR genes in Fayette lines may lead to effector recognition and activation of downstream responses which can, in turn, affect the level and sensitivity of SCN resistance. It is worth noting that few differentially expressed NBS-LRR genes found in Fayette lines overlapped with early-response genes activated at 8 hpi, implying these NBS-LRR genes might not be directly involved in the SCN response at early stages of infection.

### The continuous induction of phytoalexin biosynthesis-related genes at early stages of infection may enhance Rhg1-mediated SCN resistance

Genes related to oxidation-reduction processes (e.g. P450 and peroxidase genes) were clustered in cluster 4, and their expression levels tended to be higher in Fayette lines with higher copy number of the repeat at the *Rhg1* locus. About half of the SCN-responsive genes at the early infection stage were identified in this cluster. Consistently, Guttikonda *et al*. (2010) examined expression profiles of P450 genes in soybean using 99 microarray libraries in public databases, and found several P450 genes were induced by SCN (e.g. *CYP736A28*, *CYP93E1*, *CYP82A4*, *CYP94C18*, and *CYP81E11*). The time-course expression profiles validated the RNAseq result that the induction of the overlapped genes in cluster 4 was found in SCN-infected roots at the migration phase (8 hpi) and remained up-regulated until the syncytium establishment stage (48 hpi) in resistant Fayette#99. On the contrary, these genes were induced at only 8 hpi in susceptible Williams 82, suggesting that this group of genes play an important role in early defense responses in both resistant and susceptible reactions, but that a more stable and sustained induction enhances *Rhg1*-mediated SCN-resistance in resistant Fayette lines. Based on protein sequence similarity, their roles were predicted as being involved in biosynthesis of secondary metabolites, especially the pathways of phenylpropanoid biosynthesis. For example, peroxidase genes involved in lignin biosynthesis and five P450 genes (e.g. two *CYP81E*, two *CYP71D8*, and one *CYP93A1*) participated in flavonoid/isoflavonoid biosynthesis toward phytoalexins, which have been reported as an insect-induced defense response gene in *Medicago* (Liu *et al*. 2003), elicitor early responsive genes in soybean (Schopfer and Ebel 1998), and a defense response marker in soybean (Kinzler *et al*. 2016), respectively. Assuming the continuous induction of these genes holds true for other Fayette lines with different expression levels correlated to the copy number, the results imply different rates of phytoalexin accumulation in SCN-infected roots of Fayette lines with CNV at the *Rhg1* locus, in turn implying that phytoalexin levels are either a mechanism of SCN resistance or are induced as part of a generalized defense response.

### Ethylene is a key hormone regulating early response genes in SCN resistance

Ethylene plays a role in modulating root colonization of cyst nematodes (Wubben *et al*. 2001; Bent *et al*. 2006), attractiveness of roots to SCN (Hu *et al*. 2017), and throughout much of the SCN life cycle in infected roots (Tucker *et al*. 2010). The AP2/ERF is another key TF family in ethylene signaling pathway involved in plant-cyst nematode interactions such as *RAP2.3* (Hermsmeier *et al*. 2000), *GmEREBP1* (Mazarei *et al*. 2002) and RAP2.6 (Ali *et al*. 2013). In this study, one AP2/ERF gene (*Glyma.02G016100*; homologous to ERF71/HRE2 in *Arabidopsis*) was identified as a bridging gene between clusters 1 and 2. The ERF71/HRE2 gene plays a role in low oxygen signaling in *Arabidopsis* (Licausi *et al*. 2010) and is regulated by ethylene, validated by the functional characterization of its closely related gene, *ERF73/HRE1* (Hess *et al*. 2011). The roles of *ERF71* and *ERF73* genes may regulate ROS homeostasis (Yao *et al*. 2017), suggesting a possible function of *Glyma.02G016100* gene in response to SCN infection; however, further analysis of this gene is still needed. It is unclear whether the ethylene responses are directly tied to resistance or are perhaps induced by root damage, given the implication of other hormone systems in SCN resistance signaling using different methods (Dong and Hudson 2022). The enrichment analysis of TF binding motifs in the promoters of DEGs illustrated three TF family binding sites (for ERFs, NACs, and HD-ZIPs), that were all more frequently identified in the promoters of DEGs in cluster 4 than in other clusters. This suggests that the ERFs, together with NACs and HD-ZIPs, may preferentially regulate genes related to oxidation-reduction processes in *Rhg1*-mediated resistance to SCN. In addition, seven AP2/ERF genes, as well as other genes related to ethylene biosynthesis and signaling, were identified as DEGs in the co-expression network. These results suggest that ethylene signaling may play an important role in regulation of defense-related genes at the early stage of infection, via differential expression of TF-encoding transcripts driving differential expression of oxidation-reduction genes in cluster 4.

## Conclusion

This study demonstrated a wide range of defense-related genes involved in Rhg1-mediated resistance to SCN, by performing RNAseq analysis on isogenic lines of the Fayette cultivar that carry 9–11 copies of a 31.2 kb repeat segment at the Rhg1 locus. The predicted defense mechanisms included integration of several TF families in signaling pathways, modulation of ethylene signaling, activation of R genes in plant immunity and an accumulation of secondary metabolites such as phytoalexins for chemical defense. We also provide evidence for the participation of genes related to biosynthesis of secondary metabolites in the early response to SCN infection in resistant soybean plants. The candidate genes will be further functionally characterized to clarify their roles in Rhg1-mediated SCN resistance and may help to improve SCN resistance in PI 88788-derived cultivars.

## Supplementary Material

jkae226_Supplementary_Data

## Data Availability

The RNA-seq data sequenced and analyzed in this study have been deposited in the NCBI Sequence Read Archive (SRA). The data can be accessed under the accession number (PRJNA1113631), and include raw sequencing reads and relevant metadata. Supplemental material available at G3 online.
