# Supplementary material for: Impact of Rhg1 copy number variation on a soybean cyst nematode resistance transcriptional network
Source: G3 (Bethesda). 2024 Sep 19;14(12):jkae226. doi: 10.1093/g3journal/jkae226 (PMC11631408; doi:10.1093/g3journal/jkae226)
Supplement: jkae226_Supplementary_Data [file jkae226_supplementary_data.pdf]

Manuscript Supplementary

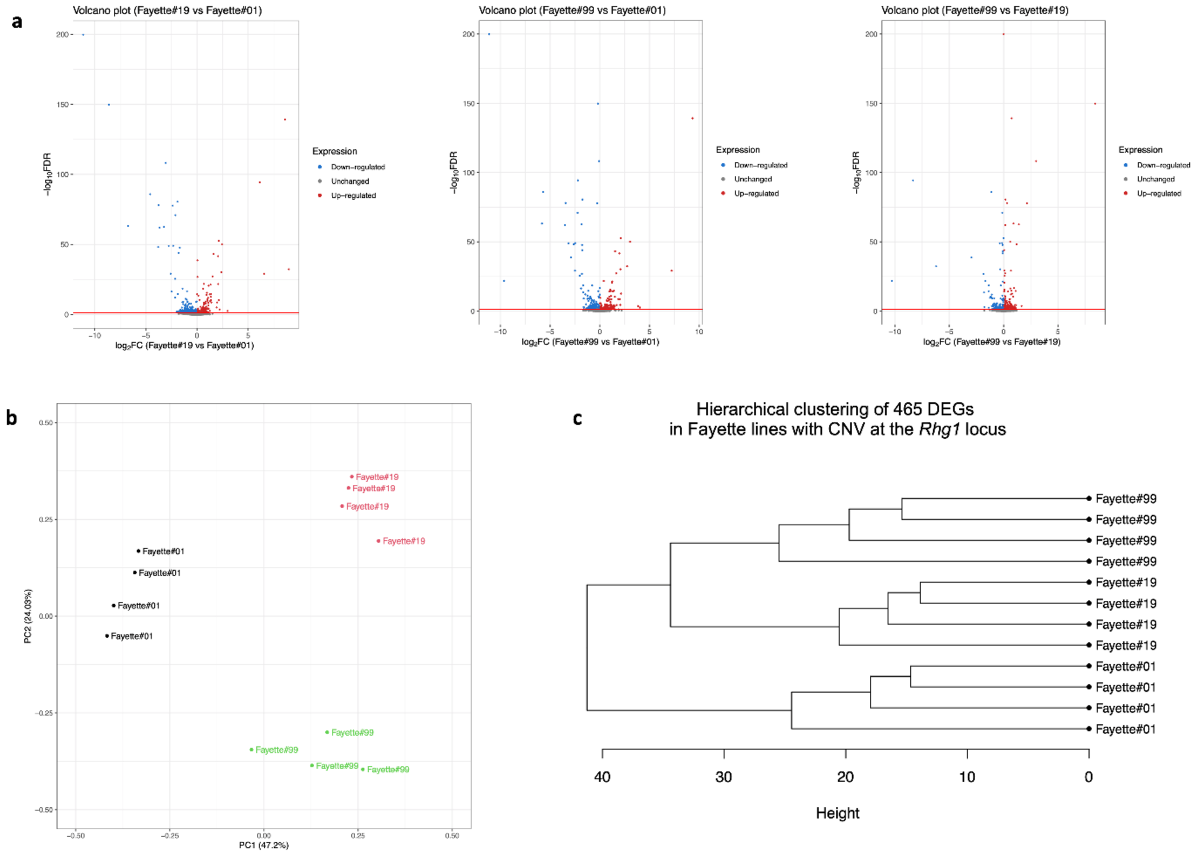

Figure S1 RNA-seq analysis of three Fayette lines (Fayette#01, Fayette#19, and Fayette#99) with copy number variation (CNV) at the *Rhg1* locus. (A) Volcano plots show differential expression with FDR cut-off set at 0.05. (B) Principal component analysis (PCA) of differentially expressed genes. (C) Hierarchical clustering of 465 differentially expressed genes (DEGs) in three Fayette lines (Fayette#01, Fayette#19, and Fayette#99) with copy number variation (CNV) at the *Rhg1* locus.

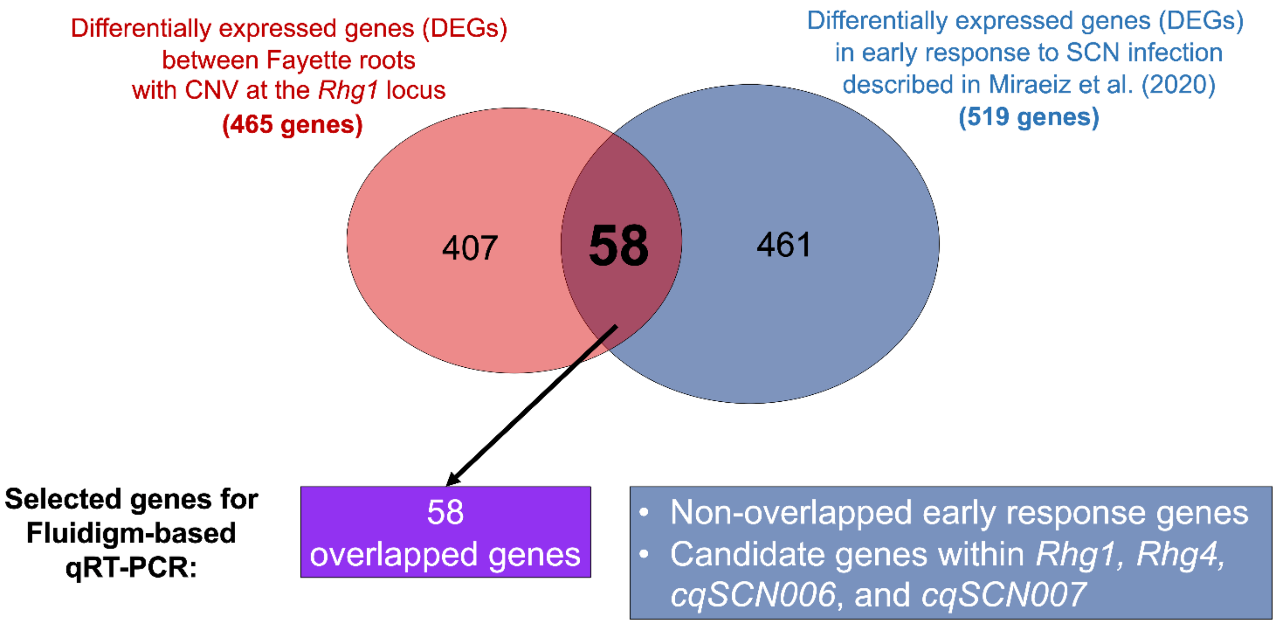

Figure S2 Venn Diagram comparing between two sets of differentially expressed genes (DEGs). Red circle represents the DEGs identified in RNA-seq analysis of three Fayette lines (Fayette#01, Fayette#19, and Fayette#99) with copy number variation (CNV) at the *Rhg1* locus. Blue circle represents the DEGs identified in RNA-seq analysis of four genotypes (Fayette#99, Peking, *Glycine soja* PI 468916, and Williams 82) in early response to SCN infection (Miraeiz et al., 2020). The rectangle represents candidate genes that were selected for qRT-PCR using Fluidigm Biomark HD system. Purple rectangle denotes overlapped genes identified in both sets of DEGs. Blue rectangle denotes non-overlapped genes that were differentially expressed in early response to SCN infection, but not differentially expressed in response to CNV at *Rhg1* in Fayette lines and genes located within *Rhg1, Rhg4, cqSCN006, and cqSCN007*.

Motif ID	Gene ID	TF family	Method	Data source	Data source ID	Motif
MP00540	Glyma.04G026300	BBR-BPC	DAP	PMID:27203113 (O'Malley et al., 2016)	AT5G42520	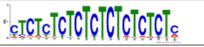
MP00253	Glyma.05G137100	BBR-BPC	DAP	PMID:27203113 (O'Malley et al., 2016)	AT2G01930	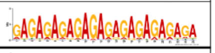
MP00126	Glyma.13G345200	bZIP	DAP	PMID:27203113 (O'Malley et al., 2016)	AT1G06070	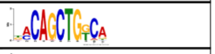
MP00229	Glyma.02G293300	C2H2	DAP	PMID:27203113 (O'Malley et al., 2016)	AT1G72050	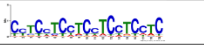
MP00118	Glyma.10G257900	C2H2	ampDAP	PMID:27203113 (O'Malley et al., 2016)	AT5G67450	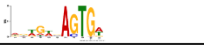
MP00043	Glyma.15G053600	CAMTA	PBM	PMID:25215497 (Weirauch et al., 2014)	M0582_1.02	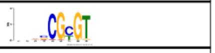
MP00623	Glyma.12G117000	ERF	PBM	PMID:25215497 (Weirauch et al., 2014)	M0004_1.02	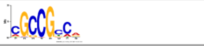
MP00311	Glyma.03G111700	ERF	DAP	PMID:27203113 (O'Malley et al., 2016)	AT2G44840	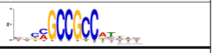
MP00486	Glyma.13G149500<br>Glyma.10G064700	HD-ZIP	DAP	PMID:27203113 (O'Malley et al., 2016)	AT5G03790	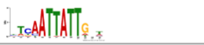
MP00323	Glyma.13G169900	HD-ZIP	DAP	PMID:27203113 (O'Malley et al., 2016)	AT3G01470	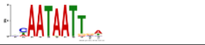
MP00322	Glyma.18G258400	HD-ZIP	DAP	PMID:27203113 (O'Malley et al., 2016)	AT3G01220	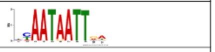
MP00320	Glyma.16G021000	HD-ZIP	DAP	PMID:27203113 (O'Malley et al., 2016)	AT2G46680	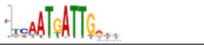
MP00225	Glyma.01G044600	HD-ZIP	DAP	PMID:27203113 (O'Malley et al., 2016)	AT1G69780	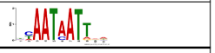
MP00510	Glyma.16G063700	MYB	DAP	PMID:27203113 (O'Malley et al., 2016)	AT5G14340	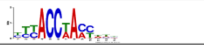
MP00479	Glyma.11G107100	MYB	DAP	PMID:27203113 (O'Malley et al., 2016)	AT4G38620	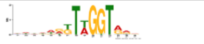
MP00438	Glyma.09G206200	MYB	DAP	PMID:27203113 (O'Malley et al., 2016)	AT4G17785	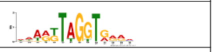
MP00337	Glyma.U027500	MYB	DAP	PMID:27203113 (O'Malley et al., 2016)	AT3G09230	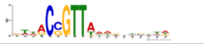
MP00259	Glyma.02G019300	MYB	DAP	PMID:27203113 (O'Malley et al., 2016)	AT2G02820	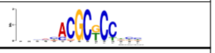
MP00612	Glyma.20G192500	NAC	ChIP-seq	SRA:SRX669382 (Zhang et al., 2015)	SRX669382	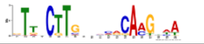
MP00439	Glyma.17G154100	NAC	DAP	PMID:27203113 (O'Malley et al., 2016)	AT4G17980	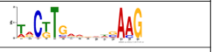
MP00363	Glyma.09G167400	NAC	DAP	PMID:27203113 (O'Malley et al., 2016)	AT3G17730	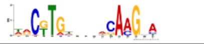
MP00343	Glyma.20G192300	NAC	DAP	PMID:27203113 (O'Malley et al., 2016)	AT3G10500	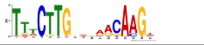
MP00333	Glyma.16G043200	NAC	DAP	PMID:27203113 (O'Malley et al., 2016)	AT3G04070	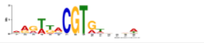
MP00179	Glyma.20G172100	NAC	DAP	PMID:27203113 (O'Malley et al., 2016)	AT1G34190	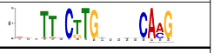
MP00004	Glyma.11G247300	Trihelix	PBM	PMID:25215497 (Weirauch et al., 2014)	M1287_1.02	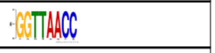
MP00618	Glyma.03G109100	WRKY	PBM	PMID:24477691 (Franco-Zorrilla et al., 2014)	WRKY12	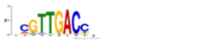
MP00539	Glyma.18G238600	WRKY	DAP	PMID:27203113 (O'Malley et al., 2016)	AT5G41570	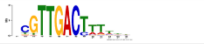
MP00531	Glyma.04G054200	WRKY	DAP	PMID:27203113 (O'Malley et al., 2016)	AT5G26170	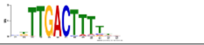
MP00525	Glyma.04G238300	WRKY	DAP	PMID:27203113 (O'Malley et al., 2016)	AT5G24110	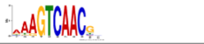
MP00408	Glyma.06G142000	WRKY	DAP	PMID:27203113 (O'Malley et al., 2016)	AT3G56400	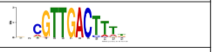
MP00094	Glyma.02G306300	WRKY	SELEX	PMID:26531826 (Mathelier et al., 2016)	MA0589.1	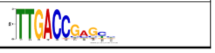

Figure S3 List of over-represented motifs and their metadata retrieved from PlantTFDB v.4. The motifs were originally retrieved from difference sources including DAP-seq (O'Malley et al., 2016), ampDAP-seq (O'Malley et al., 2016)

Table S1 Gene list and primer sequences (forward and reverse sequences) designed for qRT-PCR. Three endogenous reference genes (Bold) were used for normalization and calculation relative expression values.

**Gene List**	**Description**	**Forward Sequences**	**Reverse Sequences**
Glyma.01G114400	Otubain	CTGCTGCACTTCTTGTTCTCA	CCAGACACATGATACCACAAAATC
Glyma.01G125400	NBS-LRR	TTGAGGATCCTGGAAACCGT	GGCCTCAGTCCCCTTGTTA
Glyma.01G127100	dirigent-like gene	CCCTCATTTACGCCAATGCC	GTTGCTTCCGGTGAAGGTCT
Glyma.01G128100	WRKY33	GAAACCCAAACCCAAGGAGT	GGTGTTGTTGGAGGTGTTGT
Glyma.01G134600	Glycinol 4-dimethylallyltransferase	GTTGGTGTGTTACAAGCTGCG	GGGATAATTGCCCGGATGCC
Glyma.01G135200	CYP82A	ATCAAGCTCGGTGCCAAGAA	ATGAGCTCAGCGACGAGAAG
Glyma.01G211800	NADPH:isoflavone reductase	CTGGAGTTAAGCTGATTCAGGGA	ACGGTCTACATCCAACCCAA
Glyma.02G016200	Pollen Ole e 1 allergen and extensin family protein	AGCTTGAGAACCCCAAACCC	CGGCTCCTTGAATTGGGGAA
Glyma.02G023800	NBS-LRR	TGACATTGATCCCTCGCACG	AGTTCAGACTCCATCCTGTTGAC
Glyma.02G028100	Matrix metalloproteinase	TTCGCAATAACGACCCTCAG	CGCCGAAATGGATGATTGGTC
Glyma.02G029400	C2H2 zinc finger	CTGCATTAATTTCCCACCGCA	AGCCAAATAGCCTGAGCCTT
Glyma.02G064300	ribonuclease 1	ACCCGTAGGATACTGCAAGC	CTTATTGTCGCAATCTATAGCCGTT
Glyma.02G088700	Receptor-like kinase	CTGGCAGATAGGCTCGTGAA	CTGCTACCTGGAGTTGCACA
Glyma.02G242900	U-box domain-containing protein	CATCGAACGGATCCCCACTC	CTCACTTTCCCTTCCCCACG
Glyma.02G268200	ACC-oxidase	AATTGAGGTAATCACGAATGGGAG	TTGCTCTGTCTCCTGTGCCT
Glyma.02G307300	NAD(P)H-dependent 6'-deoxychalcone synthase	GGAAGTTCTCCTTTCCTATTGAGGT	TGCAAGGTTCATCTCCACTTGA
Glyma.03G024600	NA	AATCGAAAGCAAATGAAGCACA	GGCATGCATATTTGTGGTGTGT
Glyma.03G024900	Chitinase	CACTCCCCACCTTATCGAGC	GGTCATCTCCTCCGATGCTG
Glyma.03G038700	Peroxidase	TGGTGGATGAGATCAAAGAGGC	CCCCTAGCGCAACGACAGA
Glyma.03G042700	WRKY33	ATCCAAACCCAAGGAGTTACTACA	GCTGGTGTTGGTGTTGTTGTT
Glyma.03G044900	dirigent-like gene	GGGAGAGTGGAGGGGTTGTA	CCCAAGATCGTGATGGTGCT
Glyma.03G057500	DNAJ heat shock family protein	GGAGAAGGCTCCAGCAATGT	CCCGGCTTGATCTCAATGGT

Gene List	Description	Forward Sequences	Reverse Sequences
Glyma.03G147700	dirigent-like gene	GCAGGAAATATCACTCTTCACTCT	CAGACAAAGAAAGTGGATTTGGC
Glyma.03G157800	calmodulin	ACCGTGTCACACATTGCTCA	ATGAATCCATCAGAGTCCTTGTCA
Glyma.03G253200	Indole-3-acetic acid induced protein ARG-2 homolog	TCTCACCAGACGTGGCTACT	CACGCAAATCGGCAACATCA
Glyma.04G044900	C2H2 zinc finger	CAGAACTGCCACTCCATCGT	CCTCGGTGCATGAAGAGTGT
Glyma.04G058100	acetyltransferase	GGTCGCCTTGTCACCCAAAAAT	ACTGAACAACGAGTGGGACG
Glyma.04G121700	Polyphenol oxidase	CCGAAGGTGTAGAACCCACC	GATGAGCAGCGGATCGTACA
Glyma.04G126300	Membrane steroid binding protein 1	AGGTTGTCCAACAAGCTAAGA	GACAAACAACGCACAACTGC
Glyma.04G131100	eugenol synthase	TCCTCCGTTCCAAGCTTTCC	AGCACAGCTTTTGTATCACCG
Glyma.04G213900	alcohol dehydrogenase 1	GGAAGCCAAGGGACAGACAC	GAGATCCGTCACACCCTCAC
Glyma.04G230400	NA	CAGAACTGCTCTGTTGCTGA	GTGGTACACATGGTTCGTCG
Glyma.05G083900	NA	TAGCCTCCGAAACATTGCCT	GCACTCTTTGCACAACGGAC
Glyma.05G215900	WRKY41	TCCTTTAGCCTCACTCCTGCT	ATGAGACCCTTGGAGTGGTGA
Glyma.06G045400	C2H2 zinc finger/ZAT10	CCACAAGCATAAGCACAAGCA	CGTCCACGGTGTCTCTAAGT
Glyma.06G087800	Malic enzyme	GTGGTGGTGTGAGGGACTTG	AGCATCTCTCTCTCCCTCGG
Glyma.06G295400	NA	AGAAGATTGTGGCCTCGGTT	ACTCCTGAGTGTGAAAGAACCA
Glyma.06G303100	MYB_related CPC	GCTGACATAGATCGCTCCTT	GCAATCAAAGACCACCTCTCC
Glyma.07G023300	WRKY40	AGTGGGTTGGCTGGTTTAGA	CGCTTTCTACCTCCTTGGGAA
Glyma.07G087400	Cytosolic aldehyde dehydrogenase	AACACTCTCTACCCTGCCTC	CGTTTTTCCTGAAACAGAATCTAGG
Glyma.07G186100	putative branched-chain-amino-acid aminotransferase 7	GAGCATTGTTAGGGGTGGCA	TAGTGCACCCTTCTGGTAGC
Glyma.07G212800	Nitrite and sulphite reductase	CTGGCAACCAGTTTTGTGGG	CTCACAGGCCTAGTCACAGC
Glyma.08G021900	WRKY41	TCAAGTCTCGTTCTGGCTAAGT	TGATGCTCTTCTCTGGAAGCA
Glyma.08G091400	Glutamate decarboxylase	TGGCTGCAATCTTGGGTTCA	ACCACTTGCAGCATCCACAT
Glyma.08G108900	serine hydroxymethyltransferase	TTAACTTCGCGGTGTTCCCT	TATTTTCCAAGCGCAACGGC
Glyma.08G174900	Glutathione S-transferase	ACATTGACAAAAAGGTGTATCCTGC	CCCAAACGTGTCACCTCCATA
Glyma.08G317400	NBS-LRR-RPM1	ACAGGGGGTTTCCGAACCTA	GCACCTCTGTCAGTTCGTCA

Gene List	Description	Forward Sequences	Reverse Sequences
Glyma.09G049200	CYP81E10	CCTTGCTTTGGCCATGCTTT	CGGTCTTGCCCTACTTGTGT
Glyma.09G064200	bHLH	TCGGAATCAACGGTCATACCTT	CACCCCATGAAAACAGCAGTC
Glyma.09G087400	inositol transporter 2	GAAAGAACACACCCTGAAACCA	AGCCCCAGAAATAACTCCAGTG
Glyma.09G130400	heavy-metal-associated domain-containing protein	GCAGTATCTGGTATTTCAGGGGT	AGCTTGCCAAGACTGAATTTTGTA
Glyma.09G172100	U-box domain	AGAAGAGATGGTGTTGGGATGG	GGATTTGCCACCTTTGCGAC
Glyma.09G198900	dirigent-like gene	CAGCCAACAGGTCCTCCAAA	AGCCCGTCGAGACTAGATGT
Glyma.09G277900	Peroxidase	GTCCCAACGCTGGTTCGATT	GTAGTTCCACCAAGAGCAACG
Glyma.09G284700	Peroxidase	GAAGCGCAGTAGAGAAAGCA	CAGAACCATCACAACCCCTGA
Glyma.10G119400	Subtilisin-like serine endopeptidase family protein (Cucumisin)	GGGAGTCGTCCAATCCACAG	TCCAATCCTGCCATTCTCGC
Glyma.10G230600	heavy-metal-associated domain-containing protein	ATCCCCCTTTTCTCATTTTCCCT	AGAAACTGACTCAACCCCTGAA
Glyma.10G261200	PRp27-like protein	CAGGGTTGTGCCAAATGGTG	GCCTGACCATTCCCATTCCA
Glyma.10G295300	Glycinol 4-dimethylallyltransferase	CCATCACATGCAGACCGTTG	ACGCAAATCCAAAATGCCCG
Glyma.11G051800	CYP81E8	ATCGGCCATCTGCTTTCCTC	TACAGCGGAAGTCTCTGTGC
Glyma.11G062500	CYP71D8	GGCAACAACAGTGAAGCACA	TCCAGCAGCAAATATGTCCCAT
Glyma.11G062600	CYP71D8	GCAACGGTAGTGAAGCGGAG	CCAGCAGCAAATATGTTCCATATCA
Glyma.11G070500	NADPH:isoflavone reductase	TCAGGAGTAAATCTAATTCAGGGAG	TCGTGACGATCCACATCCAA
Glyma.11G116500	Arabidopsis protein of unknown function	TGGTTAGCTTATTAGAGAGCGTG	ATCTGCCAATTGTGAGCAACC
Glyma.11G129700	Beta-glucosidase	AGGTCCTAATTTGACTTAGAGGGT	ACCGATACACATCTTTGTTTCACC
Glyma.11G181200	F-box family protein	CCGTATCGTCCCTCATGCAC	CGGAAGGAGGAGTTGTGGAG
Glyma.11G228100	Hs1pro-1 protein	AGTTCCGACATGCCTTCCAA	TCGTAAGTGGCGCAAACAGA
Glyma.11G234300	Paralog Rhg1- WI 12	GCAACGCGGAATCTCTTTTCT	GCTTGTTGCTGGAATCAGCG
Glyma.11G234500	Paralog Rhg1-alpha-SNAP	GCATGACCCCTCTGGATTCT	AGACAGACACACAAAACAGTGTA
Glyma.11G234600	Paralog Rhg1-amino acid transporter	ATTGTTTTAAGAGATGTCCACGG	TGGCAAGCACACTTGTAACC
**Glyma.12G051100**	**F-BOX ONLY PROTEIN 3**	**GAGCCCAAGACATTGCGAGAG**	**CGGAAGCGGAAGAACTGAACC**

Gene List	Description	Forward Sequences	Reverse Sequences
Glyma.12G093100	CCR4-NOT transcription complex family protein	CAGCGAAGATCCGAATCCCA	TTGAAGGGTTTGGTGGGGTC
Glyma.12G110100	NA	CACGCTTGTACGAAACGCC	CTTGTCTTTGCCTTGCAGACC
Glyma.13G065300	NA	GATTCGCGTGTTCGTTCTCG	TGTCCGCGTACTGTTCCTTC
Glyma.13G068800	CYP82A3	GAGGAGCACCGTCAGAAGAA	CCTCCCAAAATCAATTCCAAGC
Glyma.13G302400	MYB-like 102	GGGAAACAAGTGGTCGGCTAT	TGGATTAACGTTGCCAATTGCT
Glyma.13G346700	Endochitinase PR4	TTGATCAGGCTGACTCTGGC	TCAATGTGGCAAAAATGTCCAGT
Glyma.13G370100	WRKY40	TCGTTTGCTTTCAGCAAGAGC	TCCACCTCCTTTTTGGGAAGT
Glyma.14G049000	ACC-oxidase	AGAGGAAGGCTACCCTTAACC	TGACACTGCCTCCTTGAACC
Glyma.14G049200	ACC-oxidase	TCCTAATATGCACCAACAAACACA	GGGTATTCCATGATTCACCAACTCA
Glyma.14G136300	Phytochromobilin:ferredoxin oxidoreductase	CAGTTTTACTTGTGCCTTGAGGA	CTGTAAAGGCGAAGGGATCAAG
Glyma.14G212200	U-box domain-containing protein	TTGTCCACAAGGCAAAACGC	ACCTTTCACAAGTCACACCTC
Glyma.15G012000	ABC transporter	CAAGACCAAGCATCCCCGT	TTGACCTTCAGACGTCATTGGT
Glyma.15G026400	Lipoxygenase	AGAAAAGATCCCAACAGTGAGA	ACGTCTTGAGCCACTGATTTG
Glyma.15G026500	Lipoxygenase	GGCACCGGCTTAGACTTCTT	TCCAACTTTTCCTTTCCCACCA
Glyma.15G079100	ERF	AAGCTGCACAAGTAGCACAG	TCGGTGTCCTGTAAGACAGA
Glyma.15G129200	Peroxidase	AGTTGCTTTGTCAGGTGCTCA	AGTTGGGTCACTGTTGCCAT
Glyma.15G142400	Beta-glucosidase	GGGTTTGGAGGAAAGAGGCT	GGATTCCAACCTTACACATCACT
Glyma.15G156100	CYP81E	AACAACACCACCGTAGGCTC	TCCTCGTTCGAGTTCTTGGC
Glyma.15G191200	gamma-SNAP	TCAGTCTCACGAGGTGGAGT	CCGCATCCCAAGGTGAAGAA
Glyma.16G008500	Kinase-like protein	ACCGGAAAAAGACCAAACACATATC	CATGGTTGTCAAATGGCCTGTCA
Glyma.16G064200	LRR-RLK	GTTGAGCTGATATCAATTGTGAA	TGTTTGGGGCATTGAAGACA
Glyma.16G070000	Tubby C 2	GGGGTAATACCTCTGAGTTGC	AGTTGGAGATTCACCGGCAT
Glyma.16G145600	MLO-like protein 12	TCCGAATCTTCGATTAAACCGT	TGAACCACTTTCCAATACCATGA
Glyma.16G146700	Mitochondrial phosphate transporter	ATTCCTTGATGGCTCCCGTG	GCAAGCAGCATAGAACGACG
Glyma.16G159700	NBS-LRR	TCTTCCAACCTCACAAGATGCT	CTCTTAGGCTCGGCAGATGG

Gene List	Description	Forward Sequences	Reverse Sequences
Glyma.16G162400	Tryptophan aminotransferase-related protein 4	TGCTAATGCTGGAAGTGGGG	CGGTGATTGCATTCCCAACG
Glyma.16G195600	CYP71A26	ATGGGCATGGTGATGGTGTC	CCAAACATATCCAGTATCAAAGCCT
Glyma.17G030000	PR10	TGTGAATGTGATCCAAGGTG	ATATTTGAAGGGGCTAGCTTCATTG
Glyma.17G030300	Stress-induced protein SAM22	GGCTGTTGATGCCTTCAGGA	TCTCCATCCTCAACGAAAGTGA
Glyma.17G030400	Stress-induced protein SAM22	TAGCTACAGCGTAGTGGGTG	ATTCGACAGTGAGCTTGCCA
Glyma.17G046600	Flavin-dependent monooxygenase 1	GGGAAGTCACTGGCTTTGGA	TCTGCCCCTTTCTTCTGTGG
Glyma.17G053600	calmodulin-binding family protein	CTGCGCTTGCTTTTCTCTCG	CTTCAGTTTGTCGTTCTTGTATGGT
Glyma.17G140300	Protease inhibitor/seed storage/LTP family	GGTGTTCCCAAGGGATTCGT	TGCCGGGCACAAATACTGAA
Glyma.17G245100	NA	CGGTGAACTCAGGGAGTCAG	AGATCGAACCCATGTTGCCA
Glyma.18G022400	amino acid transporter	CGGAGATGTGCTATCTGGAA	CCACTGCAAGAAGAGTTGAC
Glyma.18G022500	α-SNAP	TCGCCAAATCATGGGACAAGG	CAATGTGCAGCATCGACATGGG
Glyma.18G022700	predicted wound-inducible protein	CACTGTATGACGCCCTAAACTC	ATGGACTGCGGAACGAATC
Glyma.18G026900	RLK (putative CCR3)	ACATCTCCTTCCCCTGTCCA	ACATGTCTAGTGGCGTTGGG
Glyma.18G055600	Peroxidase	CTGTCGTAGCATTGGGTGGT	ACCCCTTGTTTGAAAAGGCAG
Glyma.18G148700	1-Deoxy-D-xylulose 5-phosphate synthase 2	TCATCAAAGCACATTGCAGCC	CCAGGTCCCAGGTCAAGATCC
Glyma.18G244500	Lecithin:cholesterol acyltransferase	CTCACAAAGCTTCTGGGGGA	GAAAGGGCAAGCCAAGCAAA
Glyma.18G244600	AP2 domain	CCCTACGGTGGTAACTCAGC	GTGTGGTGGTTGGTTCCTCA
Glyma.18G244700	Calcineurin-like phosphoesterase	GGTTGTCGGTGCTTCCTTTG	CAGTCTCGAGTCTCAAGGGTT
Glyma.18G244800	Chromatin assembly factor 1 subunit A	TCCCTCATCTCCATCATCTGAG	CCATGAAGAAAATTGTGTCTTGCG
Glyma.18G244900	p-Nitrophenyl phosphatase	CGGCTAATTATAATCGTAACCGTTC	TTTTGCCCTTTGACCGAAGC
Glyma.18G245200	LETM1-like protein	TCGAAGACGCGCATTTCCT	TTATCACCGTCGCTCATGAAAAC
Glyma.18G256900	PQQ enzyme repeat - Quinohemoprotein ethanol dehydrogenase type-1	TTCGATTCCATGCTGCCGAT	TCCATGCCCATTAGAACTTGCT
Glyma.19G076800	Lysine histidine transporter 1	GTCAGAGCTTGGATGGGGTC	CAACCACAAGCTGTTGAGGC
Glyma.19G151100	dirigent-like gene	TGACTCGAAACTTGTGGGCA	AACACTGATGGTGCTTCCGT

**Gene List**	**Description**	**Forward Sequences**	**Reverse Sequences**
Glyma.19G162200	NA	CAGAATCTGGGGTAGCTTGC	GGAAGCGATTGCATGAGAAGC
Glyma.19G245400	PR4	TGGGACGCTAGCAAACCTTA	TATTTGTCACCCGCAAGCAC
Glyma.19G254800	WRKY53	GTCAGCGTACCACTTGGACA	CCAGGATTGGGGACTTGGTG
Glyma.20G001400	Peroxidase	GGCAATAGTTAAAAGCACGGT	ATCGCAACCCCTGACAAAG
Glyma.20G126700	Cyclin	GAAAGGGAGTGATGGGTGTGA	CCTTCATCGCAGTAAAGGGC
**Glyma.20G130700**	**TIP41-like family**	**AGGATGAACTCGCTGATAATGG**	**CAGAAACGCAACAGAAGAAACC**
Glyma.20G137800	Cysteine-rich receptor-like protein kinase	GCGCTACTCTACTCTACAACCAA	GCACTGATTGCATTCCAACTCT
**Glyma.20G141600**	**Ubiquitin family**	**GTGTAATGTTGGATGTGTTCCC**	**ACACAATTGAGTTCAACACAAACCG**
Glyma.20G169200	Peroxidase	CGTGCCAATTGCTCCGTTAG	GCATGCATCGACCCAGTCA
Glyma.20G248900	Protein phosphatase 2C family protein	GCCACAGATGGGGTATGGGA	TAGCCGCCTCAACCAACATT

Data sources

PMID:27203113 (O'Malley et al., 2016)
PMID:25215497 (Weirauch et al., 2014)
SRA:SRX669382 (Zhang et al., 2015)
PMID:24477691 (Franco-Zorrilla et al., 2014)
PMID:26531826 (Mathelier et al., 2016)

Literature cited

Franco-Zorrilla JM, López-Vidriero I, Carrasco JL, Godoy M, Vera P, Solano R. 2014. DNA-binding specificities of plant transcription factors and their potential to define target genes. *Proc Natl Acad Sci USA* 111:2367–2372.

Mathelier A, Fornes O, Arenillas DJ, Chen CY, Denay G, Lee J, Shi W, Shyr C, Tan G, Worsley-Hunt R, et al. 2016. JASPAR 2016: a major expansion and update of the open-access database of transcription factor binding profiles. *Nucleic Acids Res* 44–115.

O'Malley RC, Huang SS, Song L, Lewsey MG, Bartlett A, Nery JR, Galli M, Gallavotti A, Ecker JR. 2016. Cistrome and epicistrome features shape the regulatory DNA landscape. *Cell* 165:1280–1292.

Weirauch MT, Yang A, Albu M, Cote AG, Montenegro-Montero A, Drewe P, Najafabadi HS, Lambert SA, Mann I, Cook K, et al. 2014. Determination and inference of eukaryotic transcription factor sequence specificity. *Cell* 158:1431–1443.

Zhang S, Zhou B, Kang Y, Cui X, Liu A, Deleris A, Greenberg MV, Cui X, Qiu Q, Lu F, et al. 2015. C-terminal domains of histone demethylase JMJ14 interact with a pair of NAC transcription factors to mediate specific chromatin association. *Cell Discov* 1:15003.
